# Supplementary material for: Is impaired energy production a novel insight into the pathogenesis of pyridoxine-dependent epilepsy due to biallelic variants in ALDH7A1?
Source: PLoS One. 2021 Sep 8;16(9):e0257073. doi: 10.1371/journal.pone.0257073 (PMC8425566; doi:10.1371/journal.pone.0257073)
Supplement: S1 File — (PDF) [file pone.0257073.s001.pdf]

Supplementary Tables

Supplementary Table 1: Sciex API 5000 Instrument settings for TCA cycle metabolites

	**Parent**	**Product ion**	**DP (V)**	**CE (V)**	**CXP (V)**
**Citrate**	191.0	87.0	-50	-25	-8
**$^{2}H_{4}$-citrate**	195.0	89.0	-50	-25	-8
**Isocitrate**	191.0	73.0	-50	-30	-10
**Succinate**	117.0	73.0	-25	-15	-10
**$^{13}C_{4}$-succinate**	121.0	76.0	-25	-15	-10
**Fumarate**	115.2	71.0	-100	-10	-10
**$^{13}C_{4}$-fumarate**	119.2	74.0	-100	-10	-10
**Malate**	133.0	89.0	-25	-20	-10
**$^{2}H_{3}$-L-malate**	136.0	92.0	-25	-20	-10
**Lactate**	88.7	43.0	-35	-25	-8
**^{13}C-L-lactate**	89.7	44.0	-35	-25	-8
**α-ketoglutarate**	145.1	57.0	-25	-15	-6

**$^{2}H_{4}$-α-ketoglutarate**	149.1	60.0	-25	-15	-6

Supplementary Table 2: The optimization of LC-MS/MS system

**Parameters**	**Numbers**
Dwell time	40
Curtain gas	10
Collision gas	6
Ion Spray Voltage (V)	-4500
Temperature (°C)	700
Gas 1	50
Gas 2	50

Supplementary Table 3: Primers used in the qPCR assay

**Primer ID**	**Forward primer sequence**	**Reverse primer sequence**
**ND1 (mtDNA)**	AGCCTACGCCGTACCAGTATT	GTTTCACGCCATCAGCTACTG
**ND1 (mtDNA)**	TATCGCCCTACCAATCGCAC	ATCTCTTGGGCATGGGTTCG
**aldh7a1 (nDNA)**	GCAAAAACAATGCTGACTAATGC	CTGCCGAGAGCTTTGATCTTC
**Actin (nDNA)**	GAAGATCCTGACCGAGCGTG	TGGCAAGGAACTCACCCAG

Supplementary Table 4: Lactate, electron transport chain enzyme activities and muscle biopsy histopathology and electron microscopy results of Patient 1 are summarized in supplementary table 4.

**Types of investigations**	**Investigations (reference range)**	**Results of Patient 1**
**Biochemical investigations**	Lactate (<2.4 mmol/L)	2
**Electron transport chain enzyme activity**	NADH-cytochrome c reductase (complex I+III) (1.15±0.13; range 0.53-2.72 μmol/min/g wet weight)	***0.28***
	Succinate cytochrome c reductase (complex II+III) (1.77±0.2, range 0.55-3.46 μmol/min/g wet weight)	***0.40***
	Cytochrome oxidase (complex IV) (2.95±0.28, range 1.39-6.03 μmol/min/g wet weight)	***1.06***
	Citrate synthase (6.09±0.29, range 3.48-8.03 μmol/min/g wet weight)	5.47
**Muscle histopathology, histochemistry**	Modified Gomori trichrome staining	Normal
	Electron microscopy	Normal

Abnormal results are marked italic and bold.

Supplementary Table 5: Vitamin B6 vitamers, GABA pathway and TCA cycle metabolites are depicted in Supplementary Table 5.

Supplementary Table 5A: Vitamin B6 vitamers are depicted in Supplementary Table 5A.

**Metabolites**	**PLP**	**PMP**	**PL**	**PN**	**PM**
	nM	nM	nM	nM	nM
**Samples**					
Hom d12 n=20	45	36	1.1	nd	nd
Hom d12 n=20	65	36	4.7	nd	nd
Hom d12 n=20	40	31	2.3	nd	nd
Hom d12 n=20	57	35	4.9	nd	nd
Hom d12 n=20	55	33	2.0	nd	nd
Hom d12 n=20	32	26	2.5	nd	nd
Hom d11 n=20	35	21	1.4	13	nd
Hom d11 n=20	48	37	3.2	nd	nd
Hom d11 n=20	41	26	1.8	3.4	nd
Hom d11 n=20	47	27	2.7	nd	nd
Wt d12 n=20	160	103	19	nd	nd
Wt d12 n=20	152	73	20	nd	nd
Wt d12 n=20	146	90	18	nd	nd
Wt d12 n=20	160	99	19	nd	nd
Wt d12 n=20	157	94	19	nd	nd
Wt d11 n=20	243	106	27	nd	28
Wt d11 n=20	292	139	36	31	42
Wt d11 n=20	230	96	29	nd	23
Wt d11 n=20	204	90	24	nd	22
Wt d11 n=20	282	125	45	51	37

Abbreviations: PLP= pyridoxal-5'-phosphate; PMP= pyridoxamine-5'-phosphate; PL= pyridoxal; PN= pyridoxine; PM= pyridoxamine; nd=not determined.

Supplementary Table 5B: GABA pathway and TCA cycle metabolites are depicted in Supplementary Table 5B.

Samples	GHB µmol/L	Glutamine µmol/L	Glutamate µmol/L	total GABA µmol/L	SSA µmol/L	Citrate µmol/L	Succinate µmol/L	Malate µmol/L	Fumarate µmol/L	Isocitrate µmol/L	Lactate µmol/L
WT 1	0,23	421	181	46.1	0.55	2.09	2.47	140	23.5	0.11	393
WT 2	0.15	277	208	42.8	0.67	3.95	3.48	116	15.3	0.20	430
WT 3	0.05	197	164	44.1	0.71	9.87	4.42	103	31.9	0.34	172
WT 4	0.13	282	190	44.2	0.44	5.82	6.44	140	36.3	0.25	404
WT 5	0.11	280	216	46.4	0.10	8.83	5.72	118	21.8	0.34	382
WT 6	0.04	165	147	35.5	0.48	7.62	3.28	66	14.7	0.28	154
WT 7	0.14	267	214	47.3	0.37	2.83	4.60	104	8.61	0.14	390
WT 8	0.12	245	167	46.1	0.47	4.68	4.23	107	15.2	0.19	378
WT 9	0.10	223	124	44.2	0.44	4.17	4.15	84	11.6	0.19	321
Homozygous 1	0,09*	40.0	39.5	5.6	0.02	1.14	3.47	24	2.80	0.03	48
Homozygous 2	0.01	19.9	28.2	3.24	<0.01	0.80	1.81	15	1.37	0.03	37
Homozygous 3	0.01	31.1	30.9	4.99	<0.01	0.99	1.90	18	1.89	0.04	34
Homozygous 4	0.02	35.8	33.3	6.63	<0.01	0.86	1.94	23	2.06	0.06	46
Homozygous 5	0.03	85.6	53.4	12.2	0.04	1.66	2.80	43	2.76	0.05	112
Homozygous 6	0.03	92.4	88.9	15.5	0.12	1.85	2.79	52	4.28	0.09	140
Homozygous 7	0.03	55.4	41.7	10.9	0.07	0.99	2.12	37	2.80	0.04	84
Homozygous 8	0.02	32.4	40.5	8.31	0.03	1.15	2.82	30	2.46	0.06	52

Abbreviations: WT=wildtype; GHB= gamma-hydroxybutyrate; GABA=gamma aminobutyric acid; SSA=succinic semialdehyde.

Supplementary Table 5C: Electron transport chain enzyme activities are depicted in Supplementary Table 5C.

**Electron transport chain enzymes**	**300 Homozygous whole zebrafish** nmoles/min/mg mito protein	**300 Wild Type whole zebrafish** nmoles/min/mg mito protein
NADH-Q1-Reductase (Complex I)	119.47	195.79
NADH-Cytochrome c Reductase (Rotenone Sensitive) (Complex I+III)	29.32	51.25
Succinate Cytochrome c Reductase (Complex II+III)	14.66	32.03
Cytochrome Oxidase (Complex IV)	150.25	219.42
Citrate Synthase (CS)	49.85	106.75
ATPase (Complex V)	565.88	989.24
Succinate DCIP Reductase (Complex II)	25.83	67.72
**Ratios**	**Homozygous**	**Wild type**
Complex I+III / CS =	0.59	0.48
Complex I / CS =	2.40	1.83
Complex I+III / Complex II+III =	2.00	1.60
Complex IV /CS =	3.01	2.06
Complex V /CS =	11.35	9.27

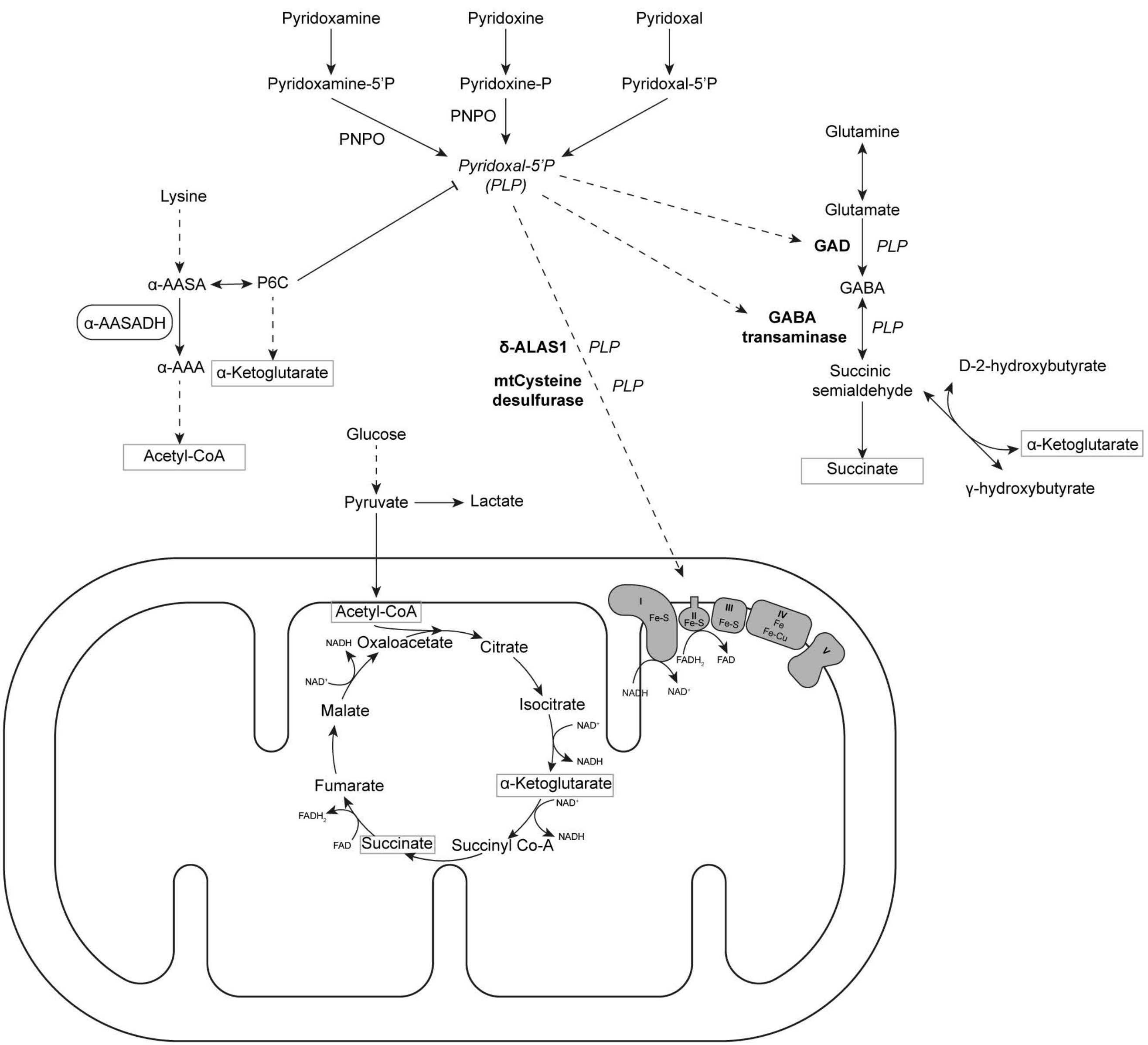

Pyridoxamine
Pyridoxine
Pyridoxal
Pyridoxamine-5'P
Pyridoxine-P
Pyridoxal-5'P
PNPO
PNPO
Pyridoxal-5'P
(PLP)
Glutamine
Glutamate
GAD
PLP
GABA
GABA transaminase
PLP
Succinic semialdehyde
D-2-hydroxybutyrate
α-Ketoglutarate
γ-hydroxybutyrate
Succinate
Lysine
α-AASA
P6C
α-AASADH
α-AAA
α-Ketoglutarate
Acetyl-CoA
δ-ALAS1
PLP
mtCysteine desulfurase
PLP
Glucose
Pyruvate
Lactate
Acetyl-CoA
Oxaloacetate
Citrate
Isocitrate
α-Ketoglutarate
Succinyl Co-A
Succinate
Fumarate
Malate
NADH
NAD+
NAD+
NADH
NAD+
NADH
FADH2
FAD
I
Fe-S
II
Fe-S
III
Fe-S
IV
Fe
Fe-Cu
V
FADH2
FAD
NADH
NAD+

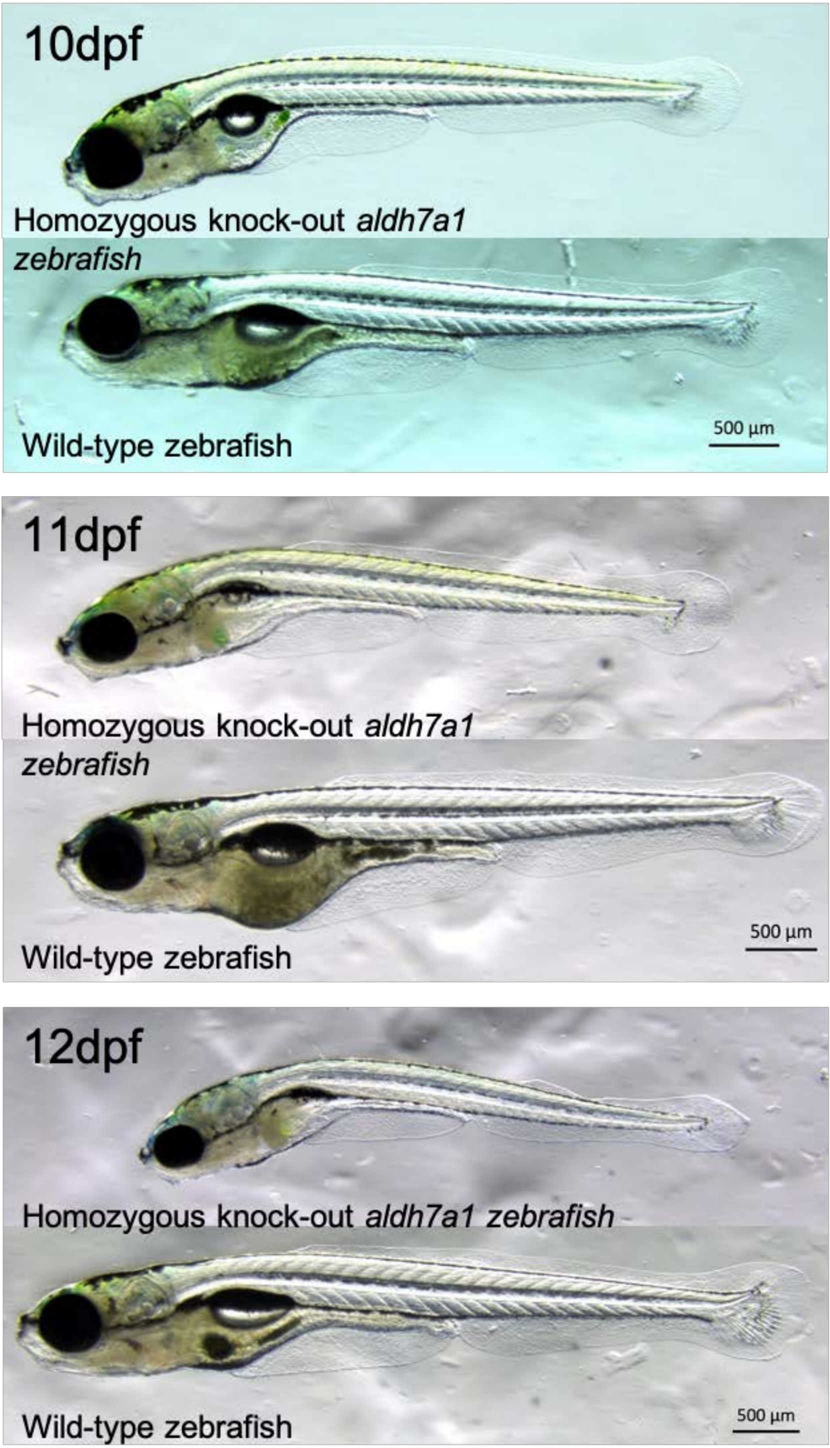
10dpf
Homozygous knock-out *aldh7a1* *zebrafish*
Wild-type zebrafish
500 µm
11dpf
Homozygous knock-out *aldh7a1* *zebrafish*
Wild-type zebrafish
500 µm
12dpf
Homozygous knock-out *aldh7a1 zebrafish*
Wild-type zebrafish
500 µm
