# Supplementary material for: Is impaired energy production a novel insight into the pathogenesis of pyridoxine-dependent epilepsy due to biallelic variants in ALDH7A1?
Source: PLoS One. 2021 Sep 8;16(9):e0257073. doi: 10.1371/journal.pone.0257073 (PMC8425566; doi:10.1371/journal.pone.0257073)
Supplement: Attachment — Submitted filename: ResponsetoReviewersEditorialBoardCleandocx.docx [file pone.0257073.s002.docx]

**Response to Reviewers and Editorial Board**

1. If you would like to make changes to your financial disclosure, please include your updated statement in your cover letter.

**Answer:** Thanks very much. We added this information into the cover letter. We hope that our responses are satisfactory to the Editorial Board.

2. Guidelines for resubmitting your figure files are available below the reviewer comments at the end of this letter. While revising your submission, please upload your figure files to the Preflight Analysis and Conversion Engine (PACE) digital diagnostic tool, <https://pacev2.apexcovantage.com/>. PACE helps ensure that figures meet PLOS requirements. To use PACE, you must first register as a user. Registration is free. Then, login and navigate to the UPLOAD tab, where you will find detailed instructions on how to use the tool. If you encounter any issues or have any questions when using PACE, please email PLOS at [figures@plos.org](mailto:figures@plos.org). Please note that Supporting Information files do not need this step.

**Answer:** Thanks very much for this information. We applied these. We hope that our responses are satisfactory to the Editorial Board.

3. If applicable, we recommend that you deposit your laboratory protocols in protocols.io to enhance the reproducibility of your results. Protocols.io assigns your protocol its own identifier (DOI) so that it can be cited independently in the future. For instructions see: <http://journals.plos.org/plosone/s/submission-guidelines#loc-laboratory-protocols>. Additionally, PLOS ONE offers an option for publishing peer-reviewed Lab Protocol articles, which describe protocols hosted on protocols.io. Read more information on sharing protocols at <https://plos.org/protocols?utm_medium=editorial-email&utm_source=authorletters&utm_campaign=protocols>.

**Answer:** Thanks very much for this information. This is not applicable for our study. We hope that our responses are satisfactory to the Editorial Board.

4. We note that you have indicated that data from this study are available upon request. PLOS only allows data to be available upon request if there are legal or ethical restrictions on sharing data publicly. For information on unacceptable data access restrictions, please see <http://journals.plos.org/plosone/s/data-availability#loc-unacceptable-data-access-restrictions>.

**Answer:** All data was included for metabolite measurements as supplemental tables during revisions process. There is no other data available. We hope that our responses are satisfactory to the Editorial Board.

5. In your revised cover letter, please address the following prompts:

a) If there are ethical or legal restrictions on sharing a de-identified data set, please explain them in detail (e.g., data contain potentially identifying or sensitive patient information) and who has imposed them (e.g., an ethics committee). Please also provide contact information for a data access committee, ethics committee, or other institutional body to which data requests may be sent.

**Answer:** Thanks very much for this information. This is not applicable for our study. We hope that our responses are satisfactory to the Editorial Board.

b) If there are no restrictions, please upload the minimal anonymized data set necessary to replicate your study findings as either Supporting Information files or to a stable, public repository and provide us with the relevant URLs, DOIs, or accession numbers. Please see <http://www.bmj.com/content/340/bmj.c181.long> for guidelines on how to de-identify and prepare clinical data for publication. For a list of acceptable repositories, please see <http://journals.plos.org/plosone/s/data-availability#loc-recommended-repositories>.

**Answer:** Thanks very much for this question. This information is included as supplemental data. We hope that our responses are satisfactory to the Editorial Board.

6. Thank you for including your ethics statement: "The study was approved by Institutional Research Ethics Board (REB#1000050808) for human participants."   

a. Please amend your current ethics statement to include the full name of the ethics committee/institutional review board(s) that approved your specific study.

**Answer:** Thanks very much for this question. This information is included. We hope that our responses are satisfactory to the Editorial Board.

b. Once you have amended this/these statement(s) in the Methods section of the manuscript, please add the same text to the “Ethics Statement” field of the submission form (via “Edit Submission”).
For additional information about PLOS ONE ethical requirements for human subjects research, please refer to <http://journals.plos.org/plosone/s/submission-guidelines#loc-human-subjects-research>.

**Answer:** Thanks very much for this question. This information is included. We hope that our responses are satisfactory to the Editorial Board.

7. In your Methods section, please provide additional information about the participant recruitment method and the demographic details of your participants. Please ensure you have provided sufficient details to replicate the analyses such as: a) the recruitment date range (month and year), b) a description of any inclusion/exclusion criteria that were applied to participant recruitment, c) a table of relevant demographic details, d) a statement as to whether your sample can be considered representative of a larger population, e) a description of how participants were recruited, and f) descriptions of where participants were recruited and where the research took place.

**Answer:** Thanks very much for this question. This information is included into the methods and discussion sections. All information is highlighted yellow. We hope that our responses are satisfactory to the Editorial Board.

8. Please provide additional details regarding participant consent. In the ethics statement in the Methods and online submission information, please ensure that you have specified (1) whether consent was informed and (2) what type you obtained (for instance, written or verbal, and if verbal, how it was documented and witnessed). If your study included minors, state whether you obtained consent from parents or guardians. If the need for consent was waived by the ethics committee, please include this information.

**Answer:** Thanks very much for these questions. This information is included into the methods and all information is highlighted yellow. We hope that our responses are satisfactory to the Editorial Board.

9. In the ethics statement in the manuscript and in the online submission form, please provide additional information about the patient records/samples used in your retrospective study, including the source of the medical records/samples analyzed in this work (e.g. hospital, institution or medical center name).

**Answer:** Thanks very much for these questions. This information is included into the methods and all information is highlighted yellow. We hope that our responses are satisfactory to the Editorial Board.

10. At this time, we request that you  please report additional details in your Methods section regarding animal care, as per our editorial guidelines:
 
(1) Please state the source of the zebrafish
(2) Please include the method of euthanasia
(3) Please describe the care received by the animals, including the frequency of monitoring and the criteria used to assess animal health and well-being, and any steps taken to minimise animal suffering.

**Answer:** Thanks very much for these questions. This information is included into the methods and all information is highlighted yellow. We hope that our responses are satisfactory to the Editorial Board.

**Reviewer #1:** 

Major comments
Introduction
The authors could elaborate on the number of reported cases and then state what metabolic tests those patients had, which would make it easier to understand.

**Answer**: Thanks very much for this question. We included these into the introduction and highlighted yellow. We hope that our responses would be satisfactory to Reviewer 1.

Materials and Methods section
Patients
The authors have noted that “All patients were previously reported for their phenotypes, genotypes and treatment outcomes”. To aid in transparency, it would be helpful if the authors documented all of the manuscripts where a specific patient has been published.

**Answer:** Thanks very much for this question. We included these into the Material and Methods, Patients and in the results and highlighted yellow. We hope that our responses would be satisfactory to Reviewer 1.

Result
Patients
—Follow-up of these three patients can be described, such as oral pyridoxine dose, seizure outcome, and neurodevelopment.

**Answer:** Thanks very much for this question. We included these into the results and highlighted yellow. We hope that our responses would be satisfactory to Reviewer 1.

—Patient 2 was also treated with lysine restriction and arginine supplementation, which should be mentioned in this paper, and whether this treatment had any effect on the detection of GABA and TCA metabolites.

**Answer:** Thanks very much for this question. The CSF samples were not collected on arginine and lysine-restricted diet, only on pyridoxine monotherapy. This is specified in results and highlighted yellow. We hope that our responses would be satisfactory to Reviewer 1.

—The genetic age of patient 3 is unknown.

**Answer:** Thanks very much for this question. The information is included and highlighted yellow. We hope that our responses would be satisfactory to Reviewer 1.

—In the result section of patient 3, the results of CSF GABA pathway and TCA cycle metabolite results prior to pyridoxine therapy were compared with the age-matched group, but the age-matched group only has one column. So, the age of the control group was 2 weeks or 7 months? Because there are several comparisons in the text.

**Answer:** Thanks very much for this question. The information is included and highlighted yellow for each metabolite in the Table 1 and 2 and age range for each metabolite was entered under the table for Table 2 and in the table for Table 1. Indeed, in Table 1 for patient 3, the reference ranges were same for prior to pyridoxine therapy and on pyridoxine therapy. We hope that our responses would be satisfactory to Reviewer 1.

Discussion
—In the discussion part, the results of GABA pathway, tricarboxylic acid cycle metabolites, electron transport chain activities and vitamin B6 vitamers were discussed. However, although the authors carried on the substantial explanation to the meaningful result, they should emphasize the important points, not just the list of possibilities.

**Answer** Thanks very much for this question. The information is included into the conclusion and highlighted yellow. We hope that our responses would be satisfactory to Reviewer 1.

—The authors classified the three reported patients into severe and mild phenotypes. It would aid the reader if the authors stated how they defined a severe and mild phenotype. And it should be stated which patients showed severe phenotype and which showed mild.

**Answer:** Thanks very much for this question. As there was no objective measurement of disease severity, we listed the specifications for each patient for their TCA, electron transport and GABA pathways. We also updated information in the abstract. All changes are highlighted yellow. We hope that our responses would be satisfactory to Reviewer 1.

—The levels of TCA cycle metabolites in patient 2 were not different from those in the age control group, which was not discussed in the discussion section.

**Answer:** Thanks very much for this question. We included information into the discussion. All changes are highlighted yellow. We hope that our responses would be satisfactory to Reviewer 1.

—Seizures in PDE are known to be caused by elevated glutamate levels and reduced GABA levels. The seizures in patients 2 and 3 were controlled, but the metabolic results showed that glutamate levels were normal in patient 2 and increased in patient 3 compared to age matched controls. This result requires additional explanation.

**Answer:** Thanks very much for this question. We included information into the discussion. All changes are highlighted yellow. We hope that our responses would be satisfactory to Reviewer 1

—In the untreated knock-out PDE zebrafish model, the levels of GABA-related metabolites, especially glutamate, are reduced, but the levels of glutamate were increased in patients with severe phenotypes, which also requires further explanation and explanation. A similar situation occurs in the levels of TCA metabolites.

**Answer:** Thanks very much for this question. We included this information into the discussion. All changes are highlighted yellow. We hope that our responses would be satisfactory to Reviewer 1

—The authors examined and compared GABA pathway metabolites and TCA cycle metabolites, electron transport chain activities and vitamin B6 vitamers in homozygous knock-out aldh7a1 zebrafish and wild-type. If so, could heterozygous zebrafish also serve as a control group.

**Answer:** Thanks very much for this question. We included this information into the discussion. All changes are highlighted yellow. We hope that our responses would be satisfactory to Reviewer 1

Minor comments
—Paragraph 12, line 251-253
“He had elevated urine α-AASA (8.8 mmol/mol creatinine; reference range 0-0.5 and compound heterozygous variants (c.500A>G (p. Asn167Ser) and c.1481+1G>T) in ALDH7A1 confirming the diagnosis of PDE-ALDH7A1 at the age of 11 years (9).” A parenthesis is missing after 0.5.

**Answer:** Thanks very much for this correction, applied and highlighted. We hope that our responses would be satisfactory to Reviewer 1

**Reviewer #2:** 

1. The authors need to include, under the materials and methods section, the deuterated internal standards and where they were procured from, needs to be included, etc.

**Answer:** Thanks very much for these questions. All these information is included into the material and methods and highlighted yellow. We hope that our responses would be satisfactory to Reviewer 2.

2. A representative chromatogram showing all of the analytes analyzed would be nice; can include in supplementary materials section

**Answer:** Thanks very much for this question. Unfortunately, we did not have any figure to submit. We hope that our responses would be satisfactory to Reviewer 2.

3. Could the authors include more information about the method used to analyze samples
What column, what flow-rate, what are the retention times of the various components
How much sample was injected, was a column guard used?
What sort of linear range was observed - see line 156

**Answer:** Thanks very much for these questions. All these information is included into the material and methods and highlighted yellow. We hope that our responses would be satisfactory to Reviewer 2.

4. The authors need to denote that they were using ESI negative ion mode and also include other parameters such as CE (collision energy), DP, CXP, temperature, etc
What linear range did the authors receive for the various analyst that were analyzed
Might want to consider a Table which can be added to Supp, Materials section
The authors need to provide sufficient information for someone to reproduce the reported work

**Answer:** Thanks very much for these questions. All these information is included into the material and methods and highlighted yellow. We hope that our responses would be satisfactory to Reviewer 2.

5. The authors discuss the metabolite AASA but don't include AASA/P6C concentrations.
Also, it is not AASA but rather PC6H which is metabolized to 6-Oxo-Pip
6-0x0-pip concentrations were not analyzed in this work, which detracts from the paper

**Answer:** Thanks very much for this comment. We previously showed biochemical abnormalities in our model and reported in 2017 (PMID: 29053735). In our current study, we confirmed that all seizing embryos were homozygous by genotyping. Genotyping was chosen as quick, high throughput and cost-effective way to apply in our research study. If we would have chosen to measure biochemical parameters additionally, we would have been using more embryos. According to our ethical responsibilities for animal welfare, scientific, legal and economic reasons, we made sure to use minimum number of animals in our study. We also did not apply biochemical confirmation as an additional method due to associated high costs. We hope that our response would be satisfactory to Reviewer 2.

6. My major concern is the lack of patient samples
Obviously it is difficult to get ahold of samples from PDE patients, they are precious

**Answer:** Thanks very much for this comment. We agree with the Reviewer 2 that more patients would have been better to support our study findings. Unfortunately, we have not identified any more patients and explained our extensive efforts for the patient cohort that we worked to find more patient in the material and methods and in the discussion. We hope that our response would be satisfactory to Reviewer 2.

7. For example, not all of the patients had the same metabolic disturbances. A few of those metabolites were outside of controls but not significantly so. Certainly subject 3 had very elevated glutamate but that was only one patient.

**Answer:** Thanks very much for this comment. We agree with the Reviewer 2 that more patients would have been better to support our study findings. Unfortunately, we have not identified any more patients and explained our extensive efforts for the patient cohort that we worked to find more patient in the material and methods and in the discussion. We hope that our response would be satisfactory to Reviewer 2.

8. I really like what authors are trying to propose, a couple of additional patient samples to support the concept would be wise.

**Answer:** Thanks very much for this comment. We agree with the Reviewer 2 that more patients would have been better to support our study findings. We think that a multicenter follow-up study would be great to initiate, however this would require grant applications and funding support. Unfortunately, we have not identified any more patients and explained our extensive efforts for the patient cohort that we worked to find more patient in the material and methods and in the discussion. We hope that our response would be satisfactory to Reviewer 2.
