# Supplementary material for: Is impaired energy production a novel insight into the pathogenesis of pyridoxine-dependent epilepsy due to biallelic variants in ALDH7A1?
Source: PLoS One. 2021 Sep 8;16(9):e0257073. doi: 10.1371/journal.pone.0257073 (PMC8425566; doi:10.1371/journal.pone.0257073)
Supplement: Attachment — Submitted filename: ResponsetoReviewersRev_2.docx [file pone.0257073.s003.docx]

**Response to Reviewers**

**Reviewer #1:** 

In introduction section, the authors described “Less than 200 patients with PDE-ALDH7A1 were reported in the literature (5-8).” However, the number of previously reported cases with ALDH7A1 mutations summarized in this paper was biased. In the latest literature, Jiao X, et al. described the number of cases currently reported carrying mutations in the ALDH7A1, which is far greater than described in this paper. (Jiao X, Gong P, Wu Y, Zhang Y, Yang Z. Analysis of the Phenotypic Variability as Well as Impact of Early Diagnosis and Treatment in Six Affected Families With ALDH7A1 Deficiency. Front Genet. 2021;12:644447, and Jiao X, Xue J, Gong P, Wu Y, Zhang Y, Jiang Y, Yang Z. Clinical and genetic features in pyridoxine-dependent epilepsy: a Chinese cohort study. Dev Med Child Neurol. 2020;62(3):315-321.).

**Answer**: Thanks very much for this question. We added the references and corrected “less than 200” to “about 200”. We appreciate that Jiao et al reviewed the literature and reported in their 2020 manuscript that there were 266 patients reported in the literature. We cited their study following the “About 200 patients with PDE-ALDH7A1 were reported in the literature.” The information is included and highlighted yellow. The two references are also included and highlighted yellow.

We respectfully disagree with the Reviewer 1 regarding the number of patients that we are reporting in our study compared to the studies that Reviewer 1 cited above. Our study is not biased for the patient numbers and cannot be compared with other centers’ patient populations. We are not reporting case series for PDE-ALDH7A1 with their clinical, biochemical and genetic features, rather reporting new findings using muscle biopsy and CSF results. The above references cannot be compared with our study results from these point of view.

The above cited studies were performed at the Peking University, Beijing, China. The population of this city is about 22 million. The population of China is about 1.41 billion. Our study is performed at the University of Toronto. The population of Toronto is about 3 million. The population of Canada is about 37 million. Looking at the population of countries and cities, the above studies regarding patient numbers are likely similar.

Additionally, we reviewed the references provided by Reviewer 1. We summarized the information below focusing the study reported by Jiao et al., Dev Med Child Neurol, 2020. The authors report 33 patients with pyridoxine-dependent epilepsy (31 patients with ALDH7A1 variants and two patients with PLPBP variants). Authors report biochemical features and genotypes of the patients, summarized below. Unfortunately based on the lack of biochemical abnormalities and/or functional characterization of missense variants, this study did not confirm the diagnosis of PDE-ALDH7A1 in the patients. We summarized below the reasons why we made this conclusion:

1. Biochemical features: Authors measured α-AASA-P6C in plasma and in urine. In the manuscript they report elevated levels of these metabolites in all 15 patients (patient 1, 3, 4, 5, 6, 9, 10, 12, 14, 15, 16, 17, 18, 19, 20) on page 315, in the paragraph starting with “Fifteen patients underwent …………”. They summarized all biochemical features in Table 1. Their reference ranges are listed below the Table 1. I will only focus on the α-AASA-P6C, as this is the primary biochemical marker for PDE-ALDH7A1 and normal pipecolic acid in urine and plasma was reported previously in PDE-ALDH7A1.
   a. Plasma α-AASA-P6C reference range 4.40–24.17 μmol/L. The 15 patients reported in Table 1 had levels ranging from 4.93-24.17 μmol/L. It seems that based on the reference ranges authors provided below Table 1, all patients had normal plasma α-AASA-P6C levels.
   b. Urine α-AASA-P6C reference range 3.22–122.00 μmol/L in Table 1. The 15 patients reported in Table 1 had levels ranging from 3.22-122. It seems that based on the reference ranges authors provided, all patients had normal urine α-AASA-P6C levels.
   c. All patients with biallelic pathogenic or likely pathogenic variants in *ALDH7A1*, even on the long-term pyridoxine therapy have elevated urine α-AASA levels. Normal urine and plasma α-AASA-P6C levels exclude the diagnosis of PDE-ALDH7A1. Normal urine and plasma α-AASA-P6C levels are the biochemical functional characterization of variants. It seems that genotypes that the authors report in their study is not supported to be pathogenic or likely pathogenic due to the normal urine and plasma α-AASA-P6C levels in 15 out of 31 patients.
2. The variants for 31 patients with PDE-ALDH7A1 are listed in supplementary table 2 (S2). The authors did not discuss in detail for the variant classification.
   a. Patient 2: has two intronic variants: c.871+5G>A (IVS9+5G>A) AND c.1008+1G>A (IVS11+1G>A). There is no metabolite measurement for this patient. Usually, the first two bases of introns are considered as pathogenic or likely pathogenic. There are no functional studies if this c.871+5G>A intronic variant will affect the function of ALDH7A1 protein. In the absence of functional study and no measurements of α-AASA-P6C levels, this genotype does not support the diagnosis of PDE-ALDH7A1.
   b. Patient 7: has two missense variants. There are no biochemical investigations for this patient. There are no functional studies of these missense variants if they will affect the function of ALDH7A1 protein. In the absence of functional studies and in the absence of measurement of α-AASA-P6C levels, this genotype does not support the diagnosis of PDE-ALDH7A1.
   c. Patient 8: has one missense variant and one splice site variant. There are no functional studies for the missense variant if they will affect the function of ALDH7A1 protein. In the absence of functional studies, and in the absence of measurement of α-AASA-P6C levels, this genotype does not support the diagnosis of PDE-ALDH7A1.
   d. Patient 13: has two missense variants. There are no functional studies for the missense variants if they will affect the function of ALDH7A1 protein. In the absence of functional studies, and in the absence of measurement of α-AASA-P6C levels, this genotype does not support the diagnosis of PDE-ALDH7A1.
   e. Patient 21: has two missense variants. There are no functional studies for the missense variants if they will affect the function of ALDH7A1 protein. In the absence of functional studies, and in the absence of measurement of α-AASA-P6C levels, this genotype does not support the diagnosis of PDE-ALDH7A1.
   f. Patient 22: has one missense variant and one splice site variant. There are no functional studies for the missense variant if this will affect the function of ALDH7A1 protein. In the absence of functional studies, and in the absence of measurement of α-AASA-P6C levels, this genotype does not support the diagnosis of PDE-ALDH7A1.
   g. Patient 23: has two missense variants. There are no functional studies for the missense variants if they will affect the function of ALDH7A1 protein. In the absence of functional studies, and in the absence of measurement of α-AASA-P6C levels, this genotype does not support the diagnosis of PDE-ALDH7A1.
   h. Patient 24: has one missense variant and one splice site variant. There are no functional studies for the missense variant if this variant will affect the function of ALDH7A1 protein. In the absence of functional studies, and in the absence of measurement of α-AASA-P6C levels, this genotype does not support the diagnosis of PDE-ALDH7A1.
   i. Patient 25: has one splice site variant and one deletion. In the absence of biochemical investigations, this genotype does not confirm the diagnosis of PDE-ALDH7A1.
   j. Patient 26: has one missense variant (homozygous). There are no functional studies for the missense variant if this missense variant will affect the function of ALDH7A1 protein. In the absence of functional studies, and in the absence of measurement of α-AASA-P6C levels, this genotype does not support the diagnosis of PDE-ALDH7A1.
   k. Patient 27: has one missense variant (homozygous). There are no functional studies for the missense variant if this missense variant will affect the function of ALDH7A1 protein. In the absence of functional studies, and in the absence of measurement of α-AASA-P6C levels, this genotype does not support the diagnosis of PDE-ALDH7A1.
   l. Patient 28: has one missense variant (homozygous). There are no functional studies for the missense variant if this missense variant will affect the function of ALDH7A1 protein. In the absence of functional studies, and in the absence of measurement of α-AASA-P6C levels, this genotype does not support the diagnosis of PDE-ALDH7A1.
   m. Patient 29: has one missense variant and one deletion. There are no functional studies for the missense variant if this missense variant will affect the function of ALDH7A1 protein. In the absence of functional studies, and in the absence of measurement of α-AASA-P6C levels, this genotype does not support the diagnosis of PDE-ALDH7A1.
   n. Patient 30: has two missense variants. There are no functional studies for the missense variants if they will affect the function of ALDH7A1 protein. In the absence of functional studies, and in the absence of measurement of α-AASA-P6C levels, this genotype does not support the diagnosis of PDE-ALDH7A1.
   o. Patient 31: has two missense variants. There are no functional studies for the missense variants if they will affect the function of ALDH7A1 protein. In the absence of functional studies, and in the absence of measurement of α-AASA-P6C levels, this genotype does not support the diagnosis of PDE-ALDH7A1.

**Reviewer #2:** 

I felt that the authors have attempted to address concerns which were raised. However, I do think it is important to point out to your readers that AASA is really not as the aldehyde, but rather the intramolecular cyclized form; J Inherit Metab Dis 2019 May;42(3):565-574. doi: 10.1002/jimd.12059. Epub 2019 Mar 11.

**Answer:** Thanks very much for this comment and question. All these information is included into the introduction and highlighted yellow. We also included reference into the reference list. We hope that our responses would be satisfactory to Reviewer 2.
